# The Physiological Mechanisms of the Sex-Based Difference in Outcomes of COVID19 Infection

**DOI:** 10.3389/fphys.2021.627260

**Published:** 2021-02-09

**Authors:** Susan Wray, Sarah Arrowsmith

**Affiliations:** Department of Women’s and Children’s Health, University of Liverpool, Liverpool, United Kingdom

**Keywords:** steroid hormones, sexual dimorphism, SARS-CoV-2, pregnancy, ACE2, hormones

## Abstract

The scale of the SARS-CoV-2 pandemic has thrust a spotlight on the sex-based differences in response to viral diseases; morbidity and mortality are greater in men than women. We outline the mechanisms by which being female offers a degree of protection from COVID19, that persists even when confounders such as comorbidities are considered. The physiological and immunological mechanisms are fascinating and range from incomplete X chromosome inactivation of immune genes, a crucial role for angiotensin converting enzyme 2 (ACE2), and regulation of both immune activity and ACE2 by sex steroids. From this flows understanding of why lung and other organs are more susceptible to COVID19 damage in men, and how their distinct immunological landscapes need to be acknowledged to guide prognosis and treatment. Pregnancy, menopause, and hormone replacement therapy bring changed hormonal environments and the need for better stratification in COVID19 studies. We end by noting clinical trials based on increasing estrogens or progesterone or anti-testosterone drugs; excellent examples of translational physiology.

## Introduction

This short review focuses on how differences in the physiology of women and men affect the outcome and survival of patients with COVID19. We first review the evidence that outcomes for females are more favorable, before considering the mechanisms and relating them to viral infection. We use the binary terms “male” and “female” so we can correctly report data in published studies, which so far have not considered if COVID19 has particular effects on trans and non-binary people.

### COVID19 Outcomes Are Worse in Males

**FIGURE 1:**
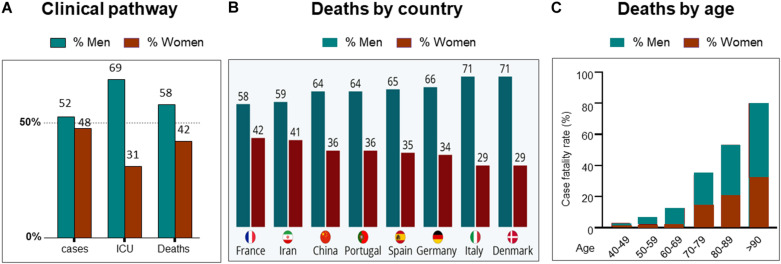
Sex differences and COVID19. **(A)** Clinical pathway from confirmed cases, intensive care unit (ICU) admissions and deaths from COVID19. Data from countries providing sex-disaggregated data in October 2020. Redrawn from Global health 5050 at https://globalhealth5050.org/wp-content/uploads/October-2020- The-COVID-19-Sex-Disaggregated-Data-Tracker-Update.pdf, accessed October 16th 2020. **(B)** Covid19 deaths by country and sex, in March 2020. Chart from Statista at: https://www.statista.com/chart/21345/coronavirus-deaths-by-gender/. **(C)** deaths from COVID19 grouped by age and sex. Data obtained from 12 countries in May 2020. Redrawn from Global health 5050.

At the time of writing (October 2020), it is almost a year since the first reports of severe acute respiratory syndrome coronavirus 2 (SARS-CoV-2), which causes coronavirus-2019 (COVID19), appeared. Since then, there has been concerted international effort to understand the virus, the disease it produces and develop strategies to combat it. From the earliest findings, it emerged that more men than women suffer severe COVID19 disease and die from it (Jin et al., 2020; Pradhan and Olsson, 2020; Scully et al., 2020). This finding of men succumbing to more severe disease and dying, was also a feature in the two previous, smaller coronavirus diseases, Middle East Respiratory System (MERS-CoV) in 2012 and SARS-CoV in 2002 (Channappanavar et al., 2017; Lu et al., 2020). For SARS-CoV-2, with its global reach and high infectivity, the continued analysis of large global data sets of sex-disaggregated data has been possible, and the data are clear; women fare better with COVID19 (Raparelli et al., 2020; Williamson et al., 2020). For updated statistical information, from ∼180 countries, the “COVID19 sex-disaggregated tracker update,” from (https://globalhealth5050.org/the-sex-gender-and-covid-19-project/) is recommended. Figure 1A is taken from their 19th October 2020 report and shows some clinical stages of COVID19 by sex. Examples of regional case fatality rates by sex can be seen in Figure 1B.

### Sex Differences Remain After Accounting for Infection Rates, Age, and Comorbidities

With the increases in available data it is possible to interrogate the statistics further and ask whether differences such as infection rates, age and co-morbidities can explain the sex differences in the outcomes of COVID19, (Abate et al., 2020). It appears that none can. Infection rates are approximately similar in women and men throughout the world (Chakravarty et al., 2020; Williamson et al., 2020)—the effects of gender and social norms, are discussed below. The effects of COVID19, and death from it, are known to increase with age. This is true for both sexes, and a variety of explanations suggested, from access to hospital and intensive care facilities, comorbidities, and immunosenescence. The latter may be due to decline in sex hormones in both sexes (Gomez et al., 2019). Menopause is specifically addressed later. When however, COVID19 deaths rates are disaggregated by age and sex, the disproportionate effect on males remains, see Figure 1C. Like age, comorbidities also worsen the progress of the disease and fatalities from it. More men die even when these factors are adjusted for. A recent study undertaken to determine who is most at risk of a severe outcome from SAR-CoV-2 infection, used a health analytical platform to obtain data from >17 million patients in the UK, within which were almost 11,000 COVID19 deaths (Williamson et al., 2020). These deaths were associated with being male, and various medical conditions, including asthma and diabetes. A multivariate analysis confirmed the sex difference in deaths, even when adjusted for all other factors, including age, obesity and diseases (diabetes, cancer, kidney, asthma, and ten others). Although beyond the scope of this review, a substantially higher death rate was found in South Asian and black people compared to white people, that was only partially attributable to comorbidities and deprivation.

While future studies will further refine our knowledge concerning disease outcomes, sex makes a significant contribution to outcomes; in COVID19, women have a degree of protection compared to men. We are not saying that all the aspects of COVID19 can be attributed to sex differences, but rather, that benefits will follow from understanding the disease better, and biological sex is a part of this. We will briefly mention how gender may impact on these data, before a more detailed discussion of the physiological mechanisms of the reported sex differences.

### Gender

There are many ways that gender can impact on COVID19 statistics. Compared to men, women may be more concerned about COVID19 (Brooks and Saad, 2020). This may lead to greater compliance with public health policies such as mask wearing, hand washing, and social distancing. In addition, globally women spend less time out of the home. These factors may reduce their infection rates, but are countered by the fact that they contribute significantly higher to the healthcare work force—an analysis of 104 countries by the World Health Organization in 2019 found that women represent around 70% of the health workforce. Men may wait longer to seek a doctor after infection and therefore be sicker before treatment. In addition, more men are, or have been, smokers, drink alcohol and have cardiovascular disease. These factors will all increase susceptibility to COVID19, but as discussed earlier, cannot explain the findings of sex differences.

## Mechanism for Sex Differences During COVID19

We first overview how SARS-CoV-2 infects humans as the basis for understanding how sex-based differences can arise. We then focus on the role of angiotensin converting enzyme 2 (ACE2) and infection, and then sex differences in immunological responses to infection.

### Overview of SARS-CoV-2

**FIGURE 2:**
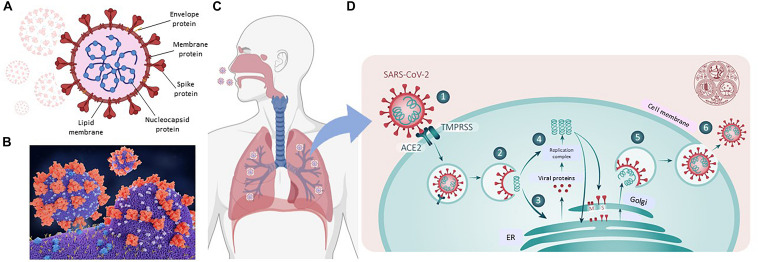
SARS-CoV-2 and COVID19. **(A)** Representation of SARS-CoV-2 virus. **(B)** A molecular model showing the virus with spike proteins (red) and ACE2 (angiotensin converting enzyme 2) receptor (blue) on host cell surface. From Juan Gaertner/Science Photo Library, accessed 8/11/2020. **(C)** Covid19 entry via airways. **(D)** scheme showing TMPRSS activation of virus, followed by its internalization, processing and replication, Adapted from Ward P. et al. (2020). Available at: https://www.fpm.org.uk/blog/covid-19-sars-cov-2-pandemic. Accessed 8/11/2020.

Coronaviruses are large-enveloped, single stranded, positive-sense RNA viruses. They contain transmembrane spike glycoproteins; composed of heads, which have host receptor binding domains and stalks, responsible for membrane fusion and infection of the host cell (Figure 2A). Compared to the 2003 SARS-CoV outbreak, SARS-CoV-2 is better able to evade our immune defenses and is highly infectious, hence the current pandemic. Both viruses use the receptor for ACE2 as their attachment target, starting in the lungs (Li et al., 2003; Tai et al., 2020; Figures 2B,C.^1^ That this is essential for viral entry was shown using ACE2 knock out mice (Kuba et al., 2005). Infection is associated with both shedding and down-regulation of the ACE2 receptor, which as discussed below, will have physiological consequences (Heurich et al., 2014; Figure 2D). The SARS-CoV-2 virus has a higher binding affinity than SARS-CoV. For infection, the stalks must be activated, and this is achieved by proteases, specifically the host cell’s transmembrane serine protease 2 (TMPRSS2, see Figure 2D; Belouzard et al., 2009). It has however been found that with SARS-CoV-2, there is an element of self-activation performed by the viral proprotein convertase furin. This facilitates SARS-CoV-2 entry, a property that it exploits in those cells that have low TMPRSS2 expression (Shang et al., 2020).

### ACE2

**FIGURE 3:**
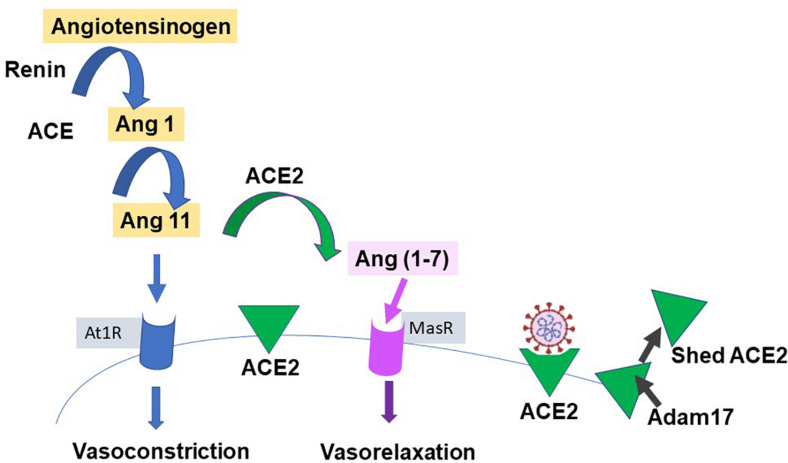
Simplified renin-angiotensin system. Scheme shows the role of ACE2 as both the catalysis for Ang(1-7) creation, with a vasodilatory effect on vascular smooth muscle, and as the receptor for SARS-CoV-2. Proteases such as those of the Adam group act to cause shedding of ACE2 into plasma.

Our knowledge of ACE2 and its relation to the classic renin-angiotensin-aldosterone system (RAAS), is relatively recent; (Donoghue et al., 2000; Tipnis et al., 2000) see Figure 3 for a simple scheme. It has been labeled the protective counter arm of RAAS as it has positive metabolic effects, and is vasodilating, anti-proliferation, and anti-inflammatory, balancing angiotensin II’s vasoconstrictive role (White et al., 2019; Samavati and Uhal, 2020).

ACE2 is a zinc containing, carboxy peptidase that removes an amino acid and converts angiotensin 1 to angiotensin 1–9 and angiotensin 11 into the vasodilator, angiotensin(1-7), and may have additional substrates (Hamming et al., 2007; Figure 3). Its catalytic site is extracellular. ACE2 is cleaved from cells by metalloproteases such as ADAM10 and ADAM17 and is shed with an active catalytic site into plasma (Turner, 2015), see Figure 3). Men appear to have higher plasma ACE2 levels than women (see Salah and Mehta, 2020). Physiologically, Ang-(1-7) has been shown to signal via a novel GPCR, Mas (Bader et al., 2018). As infection produces a down-regulation of ACE2, this may contribute to the hypertension and inflammation seen with COVID19, as the vasodilator Ang(1-7) is decreased (Povlsen et al., 2020; Samavati and Uhal, 2020), and has led to the suggestion that exogenous ACE2 could be therapeutic (Verdecchia et al., 2020). ACE2 expression can be modulated by peptides and hypoxia (Melo Junior et al., 2020), and it and TMPRSS2 are modulated by steroid hormones (Baratchian et al., 2020; Young et al., 2020), as described next.

### ACE2, TMPRSS, and Sex

Until menopause, women are relatively protected from a variety of cardiovascular risks, including high blood pressure (Reckelhoff, 2018). Part of the underlying reason for this is the effect of sex steroid hormones on RAAS, (Dalpiaz et al., 2015; Turner, 2015; Melo Junior et al., 2020). Although it seems reasonable to anticipate sex-based differences and regulation of ACE2, research is limited, especially on human tissues (Salah and Mehta, 2020; Samavati and Uhal, 2020; Song et al., 2020). With the COVID19 pandemic, attention has focused on ACE2 in the alveoli, but it has a wide tissue distribution. Specifically, it is only moderately expressed in lung, compared to kidney, heart, fat cells, and oral mucosa, and in comparable amounts to those reported in gut, bladder, brain and adrenals (Hamming et al., 2004; Zou et al., 2020). This tissue-wide distribution probably contributes to the multi organ pathologies brought on by infection. Of note with respect to COVID19, greater ACE2 expression was found in pneumocytes from men compared to women (Song et al., 2020). In differentiated human airway epithelial cells, treated either with vehicle or estradiol, the latter expressed lower levels of ACE2 mRNA (Stelzig et al., 2020) (TMPRSS2 mRNA levels were not affected). Estradiol may also positively regulates kidney, cardiac and adipose ACE2 expression (Gupte et al., 2012; Dalpiaz et al., 2015). In rats, both sexes have age-related declines in ACE2 expression, but to a greater extent in males (Xie et al., 2006). It is important to see if ACE2 transcript are translated to protein levels on the cell membrane, but it seems likely that there is sexual dimorphism in the availability of a key infectivity component, ACE2, necessary for COVID19.

*TMPRSS2* is also widely distributed and highly expressed in epithelial cells in lungs, small intestine, heart, liver, and prostate. No significant difference in TMPRSS2 expression between males and females in human lung were found (Song et al., 2020). Its transcription and activity are controlled by androgens and discussed again in the section on males and COVID19.

Both epidemiological and experimental studies have reported sex differences in the therapeutic benefits of modulators of the RAAS pathway. It was noted that “Despite these differences, RAS inhibitors are the most commonly prescribed drugs for the treatment of chronic renal disease, irrespective of sex” (Sullivan, 2008). We consider that this point remains valid for therapeutic approaches using RAAS modulating drugs during COVID19, and could skew findings if not considered (Furuhashi et al., 2020; Reynolds et al., 2020; Young et al., 2020).

## Immunological Responses and Sex Differences

### Background

During COVID19, immune cells in the lungs produce a “cytokine storm”; specifically, interleukin-6, interleukin-1β, tumor necrosis factor α, along with infiltration of chemokines, occurs. This hypercytokinemia and infiltration of monocytes and neutrophils, produces lung injury and respiratory difficulties. This pathological consequence of the immune response underlies the use of blockers of these cytokines as therapeutic approaches (Tang et al., 2020). These differences in male and female immunological activity can be related to their differing vulnerability to the disease.

Women and men differ in their physiological responses to viral diseases (Klein and Flanagan, 2016). Compared to males, females mount stronger immune responses to combat and clear viral loads (Klein, 2012). With vaccines this can lead to females producing over-exuberant responses, of both the innate and adaptive immune systems, estimated to be twice as strong as in males. This can cause increased adverse outcomes (Klein et al., 2010), as well as the increased incidence of auto-immune and inflammatory diseases found in females. Sex-dependent steroid hormones and genes, have been linked to the mechanism determining differences between the sexes in response to viral infection (Forsyth and Anguera, 2021). Many genes associated with immune responses are present on the X chromosome. Although in females one copy of these should be inactivated, there is evidence for gene imbalance, favoring females and their immunological responses to viral infections (Wang et al., 2016; Schurz et al., 2019). One example is Toll-like receptor 7 (TLR7). The gene for this receptor which senses RNA viruses such as SARS-CoV-2, is present on the X chromosome and may escape X cell inactivation (Souyris et al., 2018). All types of immune cells have estrogen and progesterone receptors which will act as transcriptional regulators. The effects of testosterone on immune responses are not as marked as those of female hormones and there will only be one copy of the X chromosome. In addition, it has been speculated that microRNAs., which act as post−transcriptional modulators of gene expression, and are also regulated by sex hormones., may also contribute to sex-based differences, especially as the X−chromosome has a particular abundance of microRNAs (see e.g., for further details Pontecorvi et al., 2020). Although too large a topic to be covered in detail here (Channappanavar et al., 2017; Jakovac, 2020), the protective effects of estrogen (and progesterone) have been attributed to (and see also Figure 5): (i) their promotion of production of anti-inflammatory cytokines (e.g., such as interleukins 4 and 10), (ii) increasing helper T cells, (iii) increasing B cells and thereby antibodies, and (iv) suppressing production of pro-inflammatory cytokines and migration of macrophages and monocytes into infected tissue (Mauvais-Jarvis et al., 2020). These protective advantages decline with age. A different but related point concerns Vitamin D, as it has been suggested that low levels of D3 may correlate with poorer infection outcomes. Estrogen may enhance vitamin D’s actions, which include reducing the cytokine storm, and in this way contribute to sex-based differences (Pagano et al., 2020). A collection of papers covering endocrinology and COVID19 was published in 2020^2^. For a comprehensive account of the endocrinological effects on the immune system recent reviews are recommended (Gadi et al., 2020; Mauvais-Jarvis et al., 2020; Young et al., 2020). Thus, we expect that the immune landscape during a SARS-CoV-2 infection will differ between men and women and make the former more vulnerable to COVID19.

Different inflammatory patterns are also thought to lead to different occurrences of cardiac arrhythmias which are also burdening patients with COVID-19. Systemic infection and inflammatory cytokines, such as IL-6, have been shown to prolong the QT-interval and alter repolarizations (Lazzerini et al.,2020a,b). Hence, sex-based differences in cytokine expression may also be attributing to differences in mortality rates via alterations in risk of life-threatening cardiac events.

### Immune Differences With COVID19

Studies of lung injury have demonstrated increased damage in male mice and ovariectomized females, which could be reduced by estradiol administration (Speyer et al., 2005). Similar protective effects of estradiol and progesterone were observed in studies of influenza-infected animals (Robinson et al., 2011; Hall et al., 2016). When considering the role of the immune system in sex based COVID19 differences, a key question is whether they are due to differences in viral load, antibody response or plasma cytokines. With SARS female mice had lower viral loads, lower inflammatory responses, reduced lung damage and death, compared to males; this protection was lost with ovariectomy or treatment with the estrogen receptor antagonist, fulvestrant (Channappanavar et al., 2017). The detailed answers to how women and men differ in their immune responses to COVID19 has been directly addressed in a recent comprehensive study (Takahashi et al., 2020). Patients with a clinical diagnosis of moderate COVID19 who were not taking immunomodulatory medicines, were studied. No sex difference was found in viral RNA concentrations. Follow up of these patients found, however, that those females with higher salivary viral load deteriorated, whereas this correlation was not found in males. Males had higher plasma levels of immune cytokines of the innate immune system such as IL-8 and IL-18. Women had more robust T cell responses, which is consistent with findings during other infections (Amadori et al., 1995). This is an important difference, as a poor T cell response was associated with poor disease outcome in men but not in women. In women but not men, worse outcome was associated with high levels of innate immune cytokines. There were some innate immune factors, such as Il-15 that increased only in females who progressed to worse disease, an association not found in males. Women could benefit more from therapies that that dampened their innate immunity responses during initial infection period.

Thus, by disaggregating patient data by sex, key differences in the immune landscapes have been identified. This heterogeneity in immune capabilities and responses helps understanding of the distinct COVID19 progression in women and men, and may be used to guide disease prognosis and sex-specific treatments (Tang et al., 2020; Ursin et al., 2020). Of relevance here also is the use of COVID19 convalescent plasma donor therapy. As males tend to have more severe COVID19, their enhanced inflammatory responses and higher B cell recruitment, and antibodies, suggests that older males may be more useful plasma donors (Klein et al., 2020). As described below, the protective effects of estrogen and progesterone (or anti-testosterone treatments) have stimulated novel treatment trials.

## Pregnancy

Pregnancy presents a unique and complex immunological scenario; the maternal immune system needs to be able to tolerate a “foreign” developing fetus whilst also protecting the mother against infections and favoring the transfer of maternal antibodies to the fetus. Elements of host defense and innate and adaptive immunity are altered during pregnancy to provide this co-operation (Racicot et al., 2014). Whilst protecting the fetus, this immune modulation could predispose pregnant women to increased susceptibility to infection from pathogens such as viruses (Robinson and Klein, 2012). Indeed, pregnant women have been shown to be disproportionately affected by respiratory illnesses, e.g., influenza (Robinson and Klein, 2012). During the MERS and SARS outbreaks, increased morbidity and higher maternal mortality rates were found (Wong et al., 2004). Hence during the current COVID19 pandemic, higher rates of mortality and disease severity were expected in pregnant women, and shielding was recommended.

The potential increased seriousness of COVID19 in pregnancy, however, has not been observed. So far, despite ACE2 being highly expressed in the placenta (Levy et al., 2008), vertical transmission to the fetus has not been seen (Chen H. et al., 2020). There is no consensus of an effect on rates of miscarriage, stillbirth or preterm birth in COVID19-infected mothers (Dubey et al., 2020; Pettirosso et al., 2020). In terms of maternal morbidity and mortality, despite the heightened severity experienced in other viral diseases (Siston et al., 2010), studies so far have not put pregnant women at any greater risk of disease severity or complications from COVID19 compared to their non-pregnant counterparts (Chen L. et al., 2020; Collin et al., 2020). How these differences relate to the specific differences between the corona viruses has not been elucidated. Epidemics can lead to resources being removed from obstetrics, maternity, and sexual health, and diverted to emergency responses, and hence increasing maternal deaths, with or without infection.

### Physiological Protective Mechanisms and COVID19 in Pregnancy

**FIGURE 4:**
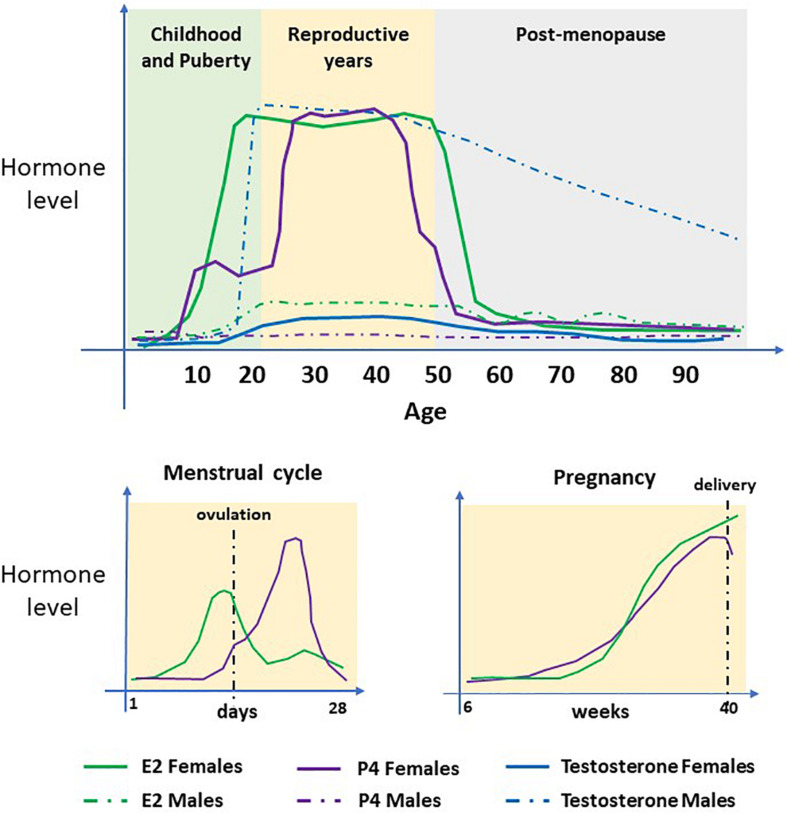
Serum concentrations of sex steroid hormones. The hormones 17β-estradiol (E2), Progesterone (P4) and Testosterone (T/DHT) in women and men during their life course. In females, E2 and P4 are the predominant hormones. Concentrations increase at puberty, undergo cyclical changes during the menstrual cycle and steadily increase during pregnancy. At menopause, concentrations decline to pre-puberty levels. T/DHT is the predominant male hormone which increases at puberty and remains high until late in life when levels decline steadily.

**FIGURE 5:**
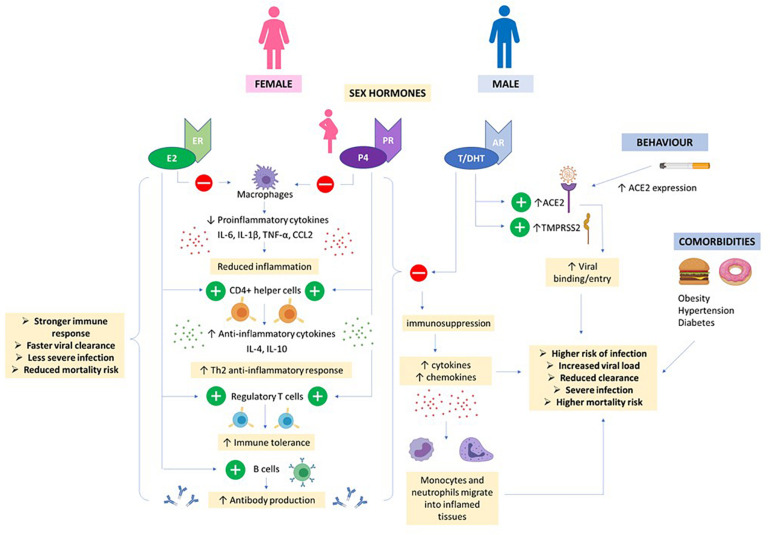
Hormones and COVID19. High E2 and P4 concentrations in females (even higher in pregnancy) helps to suppress proinflammatory cytokine production by macrophages and prevent migration of monocytes and neutrophils into inflamed tissues. CD4 + helper cells are stimulated to produce anti-inflammatory cytokines and T regulatory cells promote immune tolerance. E2 also stimulates production of antibodies. Together, this results in a stronger immune response, faster viral clearance and less severe COVID infection in women. Androgens e.g., T/DHT and AR signaling increases expression of ACE2 and TMPRSS2 promoting viral entry. Together, T/DHT’s immunosuppressive effects, male behavioral factors and co-morbidities can contribute to a more severe COVID infection and worse outcome in males.

We know from sex-based studies that females mount a greater immune response to many viral infections and this is largely due to the protective and acute effects of estrogen (Robinson et al., 2011). In pregnancy, the concentrations of 17β-estradiol (E2), estriol (E3), and progesterone are significantly increased (see Figure 4). These hormonal changes underly the immunological changes required to provide a pregnancy-supportive immune environment, as well as stimulating antibody production by B cells. Both E2 and progesterone are known to alter the number and function of multiple immune cell types producing an immunologic switch from a pro- to an anti-inflammatory state, with T-helper 2 cell dominance elevating IL-4, IL-10, IL-13, and TGF-beta (Mauvais-Jarvis et al., 2020), see Figure 5).

These changes in the hormonal milieu which shift the cytokine signature toward an anti-inflammatory state in pregnancy, may support an early adaptive immune response which helps to blunt early COVID19 infection and inflammation. In turn, this would help prevent the “cytokine storm” and its associated pulmonary pathologies, in pregnant women infected with SARS-CoV-2.

Others have suggested that folic acid supplementation during pregnancy may provide protection (Acosta-Elias and Espinosa-Tanguma, 2020). Computer simulation studies indicated that folic acid can reduce viral replication by inhibiting its furin endoprotease (Coutard et al., 2020) which is part of SARS-CoV-2 host cell entry mechanism or inhibit the coronavirus 3C-like protease, 3CL_*pro*_, (Serseg et al., 2020) required for its replication (Hsu et al., 2005). Hence the severity of infection may be inversely proportional to the concentration of folic acid but more work is required (Acosta-Elias and Espinosa-Tanguma, 2020).

As the data on COVID19 in pregnancy come from small studies and sometimes lack controls including age-matching, conclusion remains tentative but cautiously optimistic. Of note also, pregnant women may visit care settings frequently and so signs of infection may be detected and treated earlier.

## Post-Menopausal Women

That adult men of all ages and older women pose the highest risk of developing serious complications from COVID19 infection (Scully et al., 2020), again raises the question of the role of sex steroid hormones on infectivity. In women the increase in risk begins in their late 50s, see Figure 1C, around the time of the menopause (Ding et al., 2020), which is characterized by female sex hormone deficiency (Figure 4).

Animal studies of SARS-CoV and MERS, showed that absence of E2 signaling following ovariectomy or estrogen receptor antagonist treatment is associated with more severe disease in female mice (Channappanavar et al., 2017). Moreover, hormones associated with having a higher ovarian reserve (anti-Mullerian hormone and E2) negatively correlate with COVID severity (Ding et al., 2020), further suggesting that pre-menopausal women are protected.

Large-scale self-reported data obtained from the UK COVID19 symptom tracker application (C-19) showed a positive association between COVID19 and menopausal status, and a negative association with combined oral contraceptive pill use (Costeira et al., 2020), supporting the hypothesis that E2 offers protection against disease severity. Hormone replacement therapy (HRT) use, however, was positively associated with COVID19 symptoms. The route of administration, dose and type of HRT however, was not recorded and further investigations are needed (Gargaglioni and Marques, 2020). HRT is also usually only estrogenic and at physiological concentrations, whilst the combined oral contraceptive pill has E2 and progesterone and at supra-physiological concentrations.

## Men

The differences between men and women has been emphasized throughout. A few additional points can be made. Testosterone exerts immunosuppressive effects (Foo et al., 2017; Figure 5) which may contribute to a blunted antibody response in men and result in a worse prognosis compared to females (Chanana et al., 2020). Androgens, including testosterone, enhance expression of TPMRSS_2_ facilitating viral fusion with host cell membranes (Asselta et al., 2020; Hoffmann et al., 2020). Male sex hormones are also thought to increase the activity of the ACE2 receptor (Dalpiaz et al., 2015) further enabling SARS-COV-2 viral infectivity. Men with androgenetic alopecia or male pattern hair loss, a condition associated with genetic variations in the androgen receptor gene and signaling (Hillmer et al., 2005), are also thought to be at a greater risk of COVID19 severity: small studies have indicated high incidence of male pattern baldness in patients hospitalized with COVID19 (Goren et al., 2020). Along with the gender differences and detailed immunological differences reported in men with COVID19 disease discussed above, it is suggested that men will benefit from treatments that increase their T cell immune responses, and anti-testosterones.

## Clinical Trials

A SARS-CoV-2 protein interaction study mapped many potential for repurposing drugs, including sex hormones (Gordon et al., 2020). That sex hormones can modulate inflammatory responses, lessen the cytokine storm or impede viral entry, has added to the suggestion that exogenous hormones could be administered as therapies, either prophylactically or as treatment adjuncts, to reduce COVID19 disease severity. Re-purposing of existing and already approved therapies is particularly exciting given there is little time to develop new ones.

In the USA, two trials are underway testing whether symptom severity can be reduced with either a short course of estradiol, administered by transdermal patch, in adult men and older women with COVID19 (NCT04359329) or oral progesterone in men (NCT04365127). In Mexico, a trial is investigating the effect of a combined estrogen and progesterone patch (NCT04539626) on clinical response and mortality in non-severe COVID19 patients. A trial in Iran is also testing the effect of injectable estradiol and testosterone on recovery in male and female COVID19 patients with respiratory, heart or kidney failure (IRCT20150716023235N15).

Trials exploring anti-androgen therapies are also underway, including in Sweden (NCT04475601), the USA (NCT04509999, NCT04374279) and Brazil (NCT04446429) with a view to reducing disease severity in older (>50 years) male and female patients, or males presenting with male-pattern baldness, by inhibiting the expression of androgen regulated proteins, such as TMPRSS2. Other trials are investigating the effect of decreasing TMPRSS2 action using TMPRSS2 inhibitors (see Table 1).

**TABLE 1:** Clinical trial identifiers, drug class and targets.

Drug class	Target	Action/effect	Trial identifier	Sponsor/location
ER agonist	Estrogen Receptor	Increase estrogen and its effects	NCT04359329	Stony Brook University Hospital, NY, USA
ER modulator	Estrogen Receptor	Decreases estrogen production Increases testosterone production	NCT04389580*	Kafrelsheikh University Egypt
P4 hormone	Progesterone Receptor	Increase progesterone and its effects	NCT04365127	Cedars Sinai Medical Center, CA, USA
E2/P4 combined	Estrogen receptor and progesterone receptor		NCT04539626	Mexico
Anti-Androgens	Androgen Receptor	Decrease androgens/androgen signaling	NCT04374279 NCT04475601 NCT04509999 NCT04446429	Johns Hopkins, MD, USA Sweden USA Brazil
LHRH antagonist	GnRH	Decrease androgens	NCT04397718	Los Angeles, Brooklyn, Manhattan, Seattle, USA
TMPRSS2 inhibitor	TMPRSS2	Decrease TMPRSS2 action	NCT04353284 NCT04338906* NCT04374019 NCT04321096 NCT04355052* NCT04352400 NCT04355026 NCT04273763* NCT04340349*	Yale, USA Heinrich-Heine University, Germany University of Kentucky, KY, USA University of Aarhus, Denmark Sheba Medical Center, Israel University Hospital Padova, Italy General and Teaching Hospital Celje, Slovenia Wenzhou Medical University, China Instituto Nacional de Rehabilitacion, Mexico
Aldosterone antagonist	Androgen receptor	Decrease androgen signaling	NCT04345887	Istanbul University, Turkey

*Denotes trial in combination with other treatment.

In Italy, a Phase II randomized trial is planned to assess the efficacy of intravenous oxytocin in patients affected by COVID19 (NCT04386447). Oxytocin known for its role in augmenting uterine contractions in labor (Arrowsmith, 2020), has also been shown to limit excessive pro-inflammatory and oxidative stress reactions during infection by decreasing interleukin levels (Wang et al., 2015), as well as aiding nitric oxide signaling which promotes vasodilation (Thibonnier et al., 1999). Hence, oxytocin could also be used as prospective therapy for limiting COVID19 severity (Soumier and Sirigu, 2020).

### Vaccines

Passive antibody therapy for COVID19 has already been discussed (Abraham, 2020). Many vaccines are in development, in the hope of providing protection against SARS-CoV-2. From all the above it is clear that sex will be important in the immune response to such vaccines. Women will mount stronger antibody and T-cell responses and suffer worse adverse reactions. Thus, the dosage they may need of any vaccine will be less than for men. Earlier studies of the influenza vaccines have reported that the same magnitude of protective immunity is achieved by half the dose in women compared to men (Klein, 2012). If vaccine against SARS-CoV-2 is in short supply initially, would it be ethical to give smaller shots to women?

## Conclusion

Our main conclusion is that the sex-based differences in outcomes of COVID19 infection, tentatively reported at the beginning of the pandemic, have been reinforced by all subsequent studies. In addition, our understanding of the possible contributors to this is increasing but it is likely more exciting discoveries remain to be made., especially around the intersection of physiology, immunology and environmental factors.

We note that, generally, more men are enrolled in clinical trials and research in animals is often focused on males to avoid the cyclic fluctuations in hormones. This poses a significant barrier in understanding the sex-based differences in infection severity. The disparity in the effects of COVID19 observed between the sexes, and recent data in other physiological systems and pathologies, highlights the need to include both males and females in future research. There is clearly much more to be understood about sex-based differences. Understanding the mechanisms behind them may help to find appropriate and sex specific therapies for COVID19 and other sexually dimorphic pathologies (Bischof et al., 2020; Bunders and Altfeld, 2020).

## Author Notes

Biological sex affects areas of physiology and pathophysiology, beyond the obvious. Importantly these sex-based differences impact on both symptoms of disease and effectiveness of medication. In the light of the COVID19 pandemic, an appreciation of how the physiological and immunological differences between female and male responses to viral diseases is crucial, and it is to this that this review contributes. Compared to males, females mount stronger immune responses to combat and clear viral loads, but also have different immunological landscape during infection. Sex-dependent steroid hormones link mechanisms between sex and response to viral infection. For example, estrogens promote the production of anti-inflammatory cytokines, and having two X chromosomes can increase activity of immune genes carried on the chromosome, due to incomplete X-inactivation. The host viral receptor is angiotensin converting enzyme 2 (ACE2). Sex differences in ACE2’s expression in lungs and viral handling, are being actively investigated to better understand the underlying mechanisms. These differences have manifested worldwide in fewer deaths and severe COVID19 complication in females compared to males, despite roughly equal infection rates. They have also led to active clinical trials of using sex hormone treatments, such as estradiol patches, to help mitigate the effects of COVID19.

## Author Contributions

SW conceived the study. SW and SA wrote and edited the article. Both authors contributed to the article and approved the submitted version.

## Conflict of Interest

The authors declare that the research was conducted in the absence of any commercial or financial relationships that could be construed as a potential conflict of interest.

## Footnotes

1 https://scx1.b-cdn.net/csz/news/800/2020/whatistheace.jpg

2 https://www.frontiersin.org/research-topics/13975/endocrinology-and-covid–a-cross-disciplinary-topic#articles
